# Safety of surgery: quality assessment of meta-analyses on the WHO checklist

**DOI:** 10.1097/MS9.0000000000002006

**Published:** 2024-04-04

**Authors:** Mario Arturo González Mariño

**Affiliations:** Department of Obstetrics and Gynecology, Faculty of Medicine, Universidad Nacional de Colombia, Bogotá, Colombia

**Keywords:** Checklist (MeSH), general surgery, safety, World Health Organization

## Abstract

**Objectives:**

To assess the quality of the meta-analyses that review the WHO surgical safety checklist.

**Methods:**

A systematic review of meta-analysis studies was undertaken using the search terms “World Health Organization Surgical Safety Checklist” in PubMed, Embase, and Lilacs databases. The selected meta-analyses were rated using the AMSTAR 2 assessment tool.

**Results:**

In the three meta-analyses evaluated, the checklist was associated with a decrease in the rates of complications and mortality. Overall confidence in the results of the evaluated meta-analysis was critically low.

**Conclusions:**

The meta-analysis coincides with obtaining lower complications and mortality rates with the WHO surgical safety checklist. However, the studies included in the meta-analyses were mostly observational, with potential biases, and according to the AMSTAR 2 tool, the overall confidence in the results of the evaluated studies was critically low.

## Introduction

Highlights

- The WHO, seeking to get a reduction of the adverse effects of surgery, designed and implemented a checklist.
- The meta-analyses that reviewed the checklist coincide in obtaining lower complications and mortality rates.
- The evaluation using the AMSTAR 2 assessment tool showed the overall confidence in the results of the evaluated studies was critically low.

Avoidable surgical complications cause many preventable medical injuries and deaths worldwide^1^. In this situation, it is essential to recognize that it is human to make mistakes and that these mistakes may have important repercussions on patient results^2^. Failures occur because of choosing an inappropriate care method or poor performance on a care procedure considered suitable. However, errors can be reduced with training, good communication, and a “system of checks and balances”^3^. If a system to prevent errors exists and they can be remedied, patient safety in the healthcare setting will be improved^3^.

In 2008, the WHO enacted a surgical safety checklist to minimize adverse surgical events, designating a basic checklist of safety measures to be implemented globally^1^. This list is currently used in many operating rooms around the world; it is easy to apply and is improbable to cause harm^4^.

Introduction of the WHO Surgical Safety Checklist was assessed in a global setting of eight hospitals in eight countries. The results among the 3733 patients who had undergone operations before the checklist came into use were compared with those among 3955 patients whose operations took place after its use had started. Use of the list reduced any complication from 11.0 to 7.0% (*P*<0.001), with a mortality drop from 1.5 to 0.8% (*P*=0.003)^5^.

The checklist seeks to upgrade the performance of the surgical group, reduce adverse events, and improve the survival of patients after surgery^2^ through the reduction in errors such as intervening in the wrong site or patient, improving interprofessional communications, job satisfaction, and flattening of the hierarchy that often characterizes the culture of surgical teams^6^. To be effective, it appears to require an organized process of implementation, observation, and learning^7^. However, it could become a routine check-box activity that does not lead to behavioural changes, and instead, it might give an illusion of safety^8–10^. Therefore, it is important to evaluate the effects of the checklist to endorse its ongoing effort^7^.

Meta-analyses, a subset of systematic reviews, are important components of scientific information and key tools for evidence-based medicine. The number of them has been steadily growing, but not always their quality^11^. Many instruments have been designed to assess the different aspects of a systematic review. The AMSTAR 2 evaluation tool is used in this study. It allows for a more detailed evaluation of systematic reviews that also include non-randomized studies of health interventions, which are increasingly incorporated in the systematic reviews^11^.

The purpose of evaluating the meta-analyses that review the World Health Organization Surgical Safety Checklist is to assess their quality to determine the scientific support of the recommendation to implement this instrument globally.

## Methods

A search of publications was undertaken using the keywords “World Health Organization Surgical Safety Checklist” in the PubMed and Embase databases with the meta-analysis filter, without language restriction, and in Lilacs (a Latin American and Caribbean Health Sciences Literature database) with keywords in Spanish and systematic review filter, in May 2023. Reference lists of selected studies were also hand-searched to identify additional publications. The retrieved articles were reviewed by title and abstract independently with another evaluator, agreeing to read the entire article in case of discrepancy and agreement after the review. The articles selected in this screening were studied by the author with the full-text articles, determining their relevance for the review, and those that were finally extracted were scored with the AMSTAR 2 evaluation tool.

This work has been reported in line with the SQUIRE criteria.

## Results

**Figure 1:**
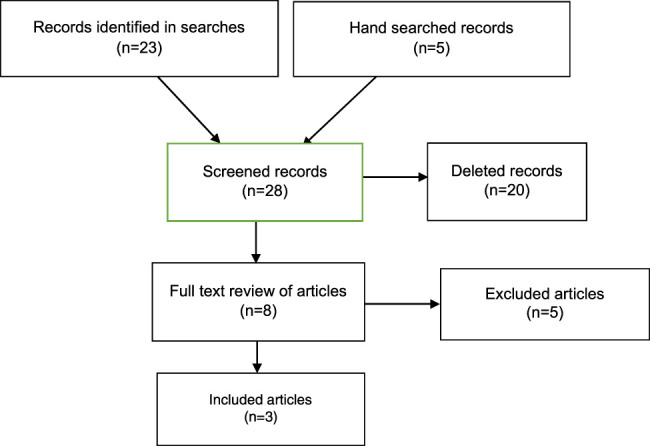
Information flow through the different phases of the systematic review.

Searches of the databases identified 23 articles and five from the reference lists of the selected studies. All of them were screened, and eight publications were selected for complete review by the author. Of these, three meta-analyzes were finally selected for qualitative analysis (Fig. 1).

Four of the full-text exclusions were because they evaluated checklists other than the WHO Surgical Safety Checklist (Biccard *et al* ^12^, Gillespie *et al* ^6^, Borchard *et al* ^13^, and Lyons *et al* ^14^). Sotto *et al* ^15^, was excluded because it was a meta-meta-analysis study.

**Figure 2:**
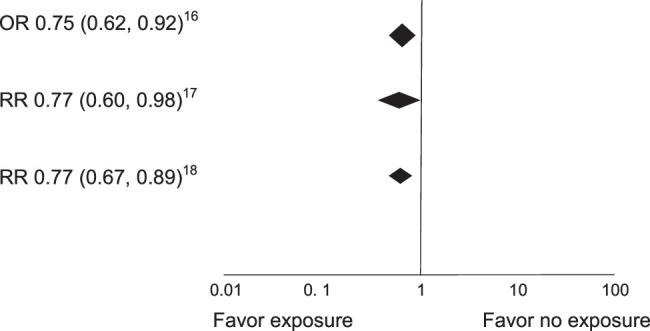
Global results of the forest plots of mortality. OR, odds ratio; RR, risk ratio.

**Figure 3:**
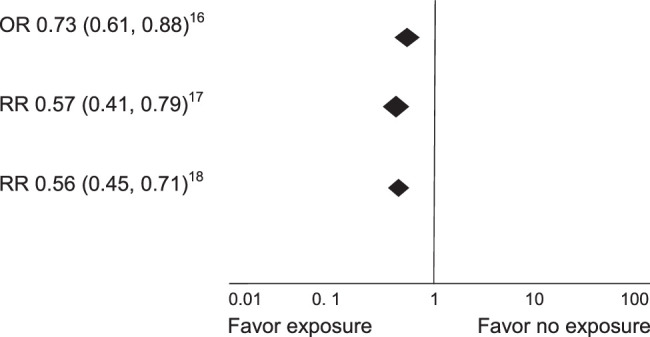
Global results of the forest plots of complications. OR, odds ratio; RR, risk ratio.

Some results of the included studies are shown (Figs. 2 and 3).

Abbott *et al* ^16^, in an analysis of 11 observational studies showed that the checklist was associated with a decrease in complication rates [odds ratio (OR) 0.73 (0.61–0.88); *P*<0.01; *I^2^* =89%] and postoperative mortality [OR 0.75 (0.62–0.92); *P*<0.01; *I^2^* =87%].

Bergs *et al* ^17^ found that seven of the 723 identified studies met the inclusion criteria (none randomized). The relative risks for any complications, mortality, and surgical site infection were 0.59 (95% confidence interval, CI: 0.47–0.74), 0.77 (0.60–0.98), and 0.57 (0.41–0.79), respectively. A strong correlation was found between a significant decrease in postoperative complications and compliance with the aspects of care included in the checklist (Q=0.82; *P*<0.01).

White *et al* ^18^, reported that the use of the WHO Surgical Safety Checklist was associated with a reduction in mortality [risk ratio (RR) 0.77; 95% CI 0.67–0.89], all complications (RR 0.56; 95% CI 0.45–0.71), and infectious complications (RR 0.44; 95% CI 0.37–0.52).

The AMSTAR 2 evaluation tool in these three meta-analyzes shows flaws in the appropriateness of meta-analytical methods for the statistical combination of results, and the assessment of the presence and likely impact of publication bias. Other particular flaws in each study result in rating the overall confidence in the results of the reviews as critically low.

## Discussion

Hospitals are at a significant risk of having adverse effects on the provision of their services. It is estimated that the incidence of these situations is ~10%, a large proportion related to surgery^17^. Given this important impact on public health, the Assembly of the WHO in 2002 enacted Resolution WHA55.18, urging member states to be attentive to the problem of patient safety. In 2004, the World Alliance for Patient Safety was approved, to facilitate patient safety policy and good practices in health care^1^. In 2008, the Alliance started the “Safe Surgery Saves Lives” strategy, the purpose of which was to improve the safety of surgery worldwide by establishing a set of safety standards to be applied universally. These standards were collected in the “WHO Surgical Safety Checklist,” divided into three stages: before, during, and after the intervention. After the launch of the campaign by the WHO, it was adopted in many nations, and the list was applied to all surgical procedures^1^.

The global introduction of the WHO surgical safety checklist aimed to improve safety in both anaesthesia and surgery and to reduce complications and mortality through better teamwork, communication, and consistency of care^19^. However, a common problem with checklists is that their introduction in clinical practice often leads to the surgical staff’s increased workload^20^, in such a way that operating room personnel may consider some steps of the list to be another bureaucratic hurdle^21^.

Some leading experts on patient safety have fostered checklists to prevent errors associated with surgery^22^. However, its effectiveness has not been defined as causal links between checklist implementation, and the results have not been well established^23^. Additionally, they are evaluated in different ways, some for compliance with the list, others for staff perceptions, complications, mortality rates, or a combination of these results^24^. Anyway, no harm has been reported with its use in patient quality or safety after the implementation of safety checklists. It is possible that studies showing no or negative effects have been performed but not published^23^.

In a meta-analysis, patients who underwent a surgical safety checklist had better postoperative results; however, this could be due to a better quality of assistance in hospitals that routinely comply with this process^16^. The other studies^17,18^ also obtained better postoperative and mortality results, but their authors expressed concerns about the limitations of the conclusions due to the quality of the studies.

The studies included in the extracted meta-analyzes were mostly observational, with potential biases, and according to AMSTAR 2, the general confidence in the results of the evaluated studies was critically low. Therefore, the meta-analyses evaluated should not be relied on to provide an accurate and comprehensive summary of the available studies^11^.

The “WHO surgical safety checklist” has been introduced in many countries; therefore, it is not advisable to conduct randomized studies with it, despite the need to establish its effectiveness. However, because the checklists must be followed and constantly evaluated as part of the safety culture, it is necessary to develop further prospective studies to establish the “WHO surgical safety checklist” utility as a long-term safety strategy^23^.

As a limitation of this study, we can mention the design of the AMSTAR 2 tool to evaluate the planning and execution of the reviews. In this tool, which includes non-randomized studies in systematic reviews, it is necessary to wait for feedback from the users of the instrument before making modifications^11^.

## Conclusions

The meta-analyses coincide in obtaining lower complications and mortality rates with the WHO surgical safety checklist. However, the studies included in the meta-analyses were mostly observational, with potential biases, and according to the AMSTAR 2 tool, the overall confidence in the results of the evaluated studies was critically low. It is important to continue with the evaluation of the WHO surgical safety checklist to improve patient safety.

## Ethical approval

The article is considered a risk-free research. This is a review whose evaluation base is published studies; people are not evaluated.

## Consent

The is a review whose evaluation base is published studies; people are not evaluated.

## Source of funding

This study did not receive external funding.

## Author contribution

The article has only one author.

## Conflicts of interest disclosure

There are no conflicts of interest.

## Research registration unique identifying number (UIN)

The study did not involve human subjects.

## Guarantor

The article has only one author.

## Data availability statement

The author confirms that any datasets generated during and/or analyzed during the current study are publicly available.

## Provenance and peer review

My paper was not invited.
